# “Hierarchy” and “Holacracy”; A Paradigm of the Hematopoietic System

**DOI:** 10.3390/cells8101138

**Published:** 2019-09-24

**Authors:** Takafumi Yokota

**Affiliations:** Department of Hematology and Oncology, Osaka University Graduate School of Medicine, Suita, Osaka 565-0871, Japan; yokotat@bldon.med.osaka-u.ac.jp

**Keywords:** hematopoietic stem cells, extramedullary hematopoiesis, HSC-niche, HSC-unit, unit networking diagram, hierarchy, holacracy

## Abstract

The mammalian hematopoietic system has long been viewed as a hierarchical paradigm in which a small number of hematopoietic stem cells (HSCs) are located at the apex. HSCs were traditionally thought to be homogeneous and quiescent in a homeostatic state. However, recent observations, through extramedullary hematopoiesis and clonal assays, have cast doubt on the validity of the conventional interpretation. A key issue is understanding the characteristics of HSCs from different viewpoints, including dynamic physics and social network theory. The aim of this literature review is to propose a new paradigm of our hematopoietic system, in which individual HSCs are actively involved.

## 1. Introduction

The population of hematopoietic stem cells (HSCs) in bone marrow has long been believed to be homogeneous and dormant at the top of the hematopoiesis hierarchy. Although this notion appears to be immiscible with the general idea of HSCs supporting life-long hematopoiesis, in which millions of diverse blood cells are continuously generated each second, the quiescent and homogeneous HSC concept has remained a doctrine firmly entrenched in the stem cell research field. This dogma also involves the concept of specific milieus-supporting HSCs, so called the “HSC niche”, of which key cellular components have been a longstanding controversial topic in this field. The scientific importance and reliability of each study involving HSCs and their niche should be considered, especially those studies carefully performed with state-of-the-art methods. However, without an updated understanding of HSC biology, how can we reconcile these different outlooks and move forward? A re-examination of the traditional theory of quiescent and homogeneous HSCs is desperately required. Then, we need to establish a comprehensive appreciation of the role HSCs play in our hematopoietic system. This review aims to describe a real paradigm of the hematopoietic system by examining recent compelling observations in extramedullary hematopoiesis and by introducing insights from biophysics and social science.

## 2. Discussion

### 2.1. Active Hematopoiesis Outside of Bone Marrow

**Figure 1:**
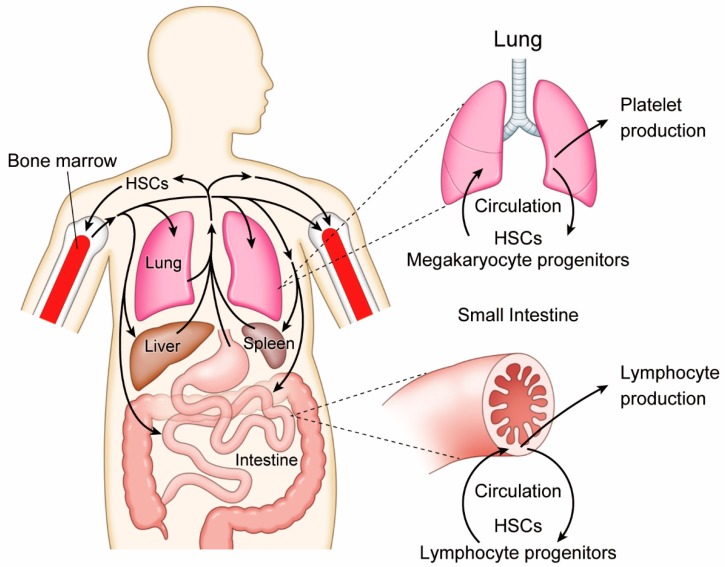
Circulating hematopoietic stem cells (HSCs) contribute to the generation of platelets in the lung and lymphocytes in the intestine.

Life-long hematopoiesis is supported by self-renewing and differentiating HSCs. Anatomical sites in which HSCs proliferate and differentiate are known to change sequentially during fetal ontogenesis [1,2]. In mouse and human, HSCs emerge in the endothelial layer of developing arteries prior to midgestation, in response to blood flow caused by cardiovascular contraction [3,4,5,6]. Then, HSCs migrate to the liver and spleen, and thereafter to the bone marrow, which is the main hematopoietic organ. HSCs are self-renewing in the bone marrow and produce a variety of blood cells throughout life. The liver and spleen can produce blood cells to compensate for bone marrow dysfunction. This reactivation of fetal hematopoietic organs is termed “extramedullary hematopoiesis”, which only occurs under pathological conditions such as severe chronic anemia or myelofibrosis. Otherwise, HSCs are believed to be preserved in a specific environment referred to as the “HSC niche” in bone marrow, where they avoid stress or age-induced attrition. Recent findings, however, have cast doubt on this conventional concept of HSCs, shedding light on a more dynamic role than ever anticipated. Indeed, accumulating data indicates HSCs actively circulate throughout the body under a homeostatic state and contribute to peripheral lympho-hematopoiesis (see below; Figure 1).

### 2.2. Lung Megakaryopoiesis

A long-standing enigma of hematology is the mechanism of mature platelet cell origins and their introduction into blood flow. Early studies determined a substantial number of megakaryocytes harbor in the lung via the pulmonary artery and release platelets from the capillary bed of this organ [7,8]. Recent advances in imaging technology using intravital microscopy have provided more direct evidence of active platelet production in the lung [9]. Indeed, it has been estimated that more than 10 million platelets are produced per hour in steady-state lungs of adult mice, which account for approximately half of total platelet production in the whole body. In the lung, turbulent blood flow caused by respiratory movement might create an ideal environment for megakaryocytes to shed platelets. This interpretation seems to be paradoxically supported by a recent success of robust platelet biogenesis ex vivo in turbulence-controlled bioreactors from induced pluripotent stem cell-derived megakaryocytes [10].

It is noteworthy that, besides mature megakaryocytes, the lung contains HSCs and megakaryocyte progenitors [9] (Figure 1). When the thrombopoietin receptor c-Mpl-deficient mice were used as recipients of lung transplantation, multi-lineage hematopoiesis, as well as long-term platelet production, were reconstituted by lung-resident hematopoietic cells. Furthermore, the HSC compartment in the bone marrow of c-Mpl-deficient recipients was also reconstituted, suggesting that HSCs in the lung are mobile and capable of relocating to other organs. Although conditions of compromised hematopoiesis in the c-Mpl-deficient bone marrow might have non-specific stimulant signals, it is indisputable that functional HSCs are residing in the respiratory organ. Still, it has yet to be determined whether HSCs are capable of self-renewal and differentiation into mature megakaryocyte in the lung.

### 2.3. Intestinal Lymphopoiesis

The gastrointestinal tract is known to contain numerous immune cells, which contribute to the development of immunological tolerance toward avirulent, commensal bacteria, as well as serve as the first line barrier against pathogenetic microorganisms. This delicate immunological balance is maintained by “gut-associated lymphatic tissue”, where immune cells migrating via peripheral circulation serve with a subset of T lymphocytes locally generated in the intestine [11,12]. B lymphocytes are also involved in the homeostatic growth of intestinal epithelial cells [13]. The local generation of lymphocytes in situ requires the existence of their progenitors as a premise. Indeed, early studies identified lymphopoietic progenitors developing in the mouse intestinal mucosa [14,15].

Likewise, the human intestine also contains HSCs with self-renewing and multilineage potentials. An early study depicted the existence of CD34⁺ cells among a CD45⁺ cell population in the epithelium and lamina propria of adult human small intestine at higher frequency than in bone marrow [16]. Those intestinal CD34⁺ CD45⁺ cells seemed to be skewed or directed toward lymphoid lineage because many of them co-expressed CD45RA, CD7, and CD56, but not the myeloid marker CD33. A recent study has supported the finding and further proved the functional integrity of intestinal HSCs through a longitudinal study of human intestinal transplantation recipients [17] (Figure 1). Intestinal HSCs in allografts are likely to contribute to immune tolerance by migrating into recipient thymus and undergoing selection in the host environment.

Intestinal HSCs do not seem to self-renew and produce lymphocytes permanently in situ because they are gradually replaced by circulating HSCs [17]. However, in the short term, it is highly likely that intestinal hematopoietic stem/progenitor cells actively participate in mucosal immunity by differentiating to lymphocytes, because IL-7 and IL15, key cytokines inducing early lymphoid differentiation are produced in the intestinal epithelium [18,19].

### 2.4. Contribution of Circulating HSCs to Myeloid Lineage in the Periphery

Activities of HSCs in the lung and intestine discussed above suggest that HSCs do not merely reside in bone marrow under intimate association with the so-called “niche”, but also dynamically participate in lympho-hematopoiesis throughout the body. Indeed, HSCs mobilized into circulating blood have long been used for transplantation in a clinical setting, and molecular mechanisms enhancing HSC mobilization have been extensively studied [20,21]. Additionally, the migration of HSCs from bone marrow to circulating blood is a recurring phenomenon under homeostatic conditions, as demonstrated in early experiments using parabiotic mice [22].

Massberg et al. identified lymphatic vessels and the thoracic duct as a major route of circulating HSCs to return to the bone marrow [23]. In mice, before entering into lymphatic vessels, circulating HSCs appear to harbor in peripheral tissues for at least 36 h, where the cells can respond to various differentiation signals. HSCs in bone marrow are known to express Toll-like receptors and their co-receptors, by which the cells can recognize bacterial lipopolysaccharide [24,25]. Likewise, circulating HSCs in thoracic duct respond to lipopolysaccharide in vivo as well as in vitro, and immediately generate myeloid cells, dendritic cells in particular, in the periphery [23]. These findings suggest the differentiation ability of HSCs is not fixed in a traditional hierarchy but is rather flexible. This flexibility allows HSCs to promptly quench local inflammation by producing innate immune cells at the site of infection.

### 2.5. Diversity and Fluctuation in the HSC Compartment

Contrary to the traditional homogeneous HSC concept, accumulating evidence has shown that the HSC population is essentially heterogeneous, composed of diverse types of cells whose self-renewing and differentiation potential differ substantially [26,27,28,29,30,31]. Elegant techniques which have genetically marked target cells, have enabled us to evaluate clonal dynamics of HSCs in vivo without transplantation. Studies with such sophisticated strategies have switched or enlarged the notion of homeostatic HSCs from classically defined long-term HSCs to long-lived progenitors [32,33]. That is, while conventional transplantation experiments demonstrated that a very small number of multipotent and self-renewing HSCs, of which phenotypes were determined as lineage makers⁻ c-kit^High^ Sca1⁺ Flt3⁻ CD48⁻ CD150⁺ ESAM⁺ EPCR⁺ in mouse and lineage makers CD34⁺ CD38⁻^/Low^ ESAM⁺ CD90/Thy1⁺ CD45RA⁻ CD49f⁺ in human [34,35,36,37,38,39,40] which reconstitute the hematopoietic system, the contribution of those long-term HSCs to physiological hematopoiesis is minimal. Instead, short-term HSCs and multipotent progenitors primarily sustain adult hematopoiesis. Furthermore, the contribution of each HSC or long-lived progenitor clone to steady-state hematopoiesis is successive, suggesting there is temporal diversity in the HSC population. Thus, life-long hematopoiesis is likely maintained by a larger variety of HSC clones than ever anticipated.

If most HSC clones were truly hibernating, how could they sustain the dynamic equilibrium of homeostatic hematopoiesis and exert the immediate apposite responses in emergent hematopoiesis? Since HSCs immediately switch between quiescent and dynamic states according to physiological demand [41,42], the ductility and durability of the hematopoietic system is likely accomplished by the integrated action of diverse HSC clones. In addition, a compelling biophysics approach has shed light on chaotic oscillations in gene expression as a necessary condition for stem cells to maintain their robust proliferation and differentiation potential [43,44]. That is, computing simulations with a dynamic-systems approach have predicted that cells executing both self-renewal and differentiation should represent temporal fluctuation or oscillation in the gene expression at the clonal level, which underlies stem cell heterogeneity [45]. Thus, the fluctuation in gene expression is considered an essential attribute, inherent to the HSC biology. Moreover, HSCs are actively transcribing a subset of genes to maintain the essence of stemness, even when they stay in quiescent cell cycle and inactive metabolism [46].

Additionally, recent studies with clonal analysis have shown that each HSC clone autonomously behaves or fluctuates in its own trajectory, determined by epigenetic configuration as well as transcriptional regulation [47,48]. Tracking individual HSCs with specific markers for 16 weeks after transplantation has revealed divergent HSC behaviors. Proliferation and lineage-bias are largely determined by the extent of DNA methylation on enhancers of critical genes at a clonal level [47]. In the same report, however, longitudinal analysis of hematopoietic dynamics under homeostatic conditions revealed that a few HSC clones were persistent for 10 months, among which some were durable whereas others were variable over time. The diversity among HSCs can be partly attributed to the difference in chromatin structures and histone modifications. Special AT-rich binding protein 1 (SATB1) is known as a global chromatin organizer that orchestrates the expression of various genes of hematopoietic progenitors as well as lymphocytes [49,50,51]. A recent study using mice in which physiological expression of SATB1 was monitored showed the HSC compartment consists of multiple cells with various SATB1 expression levels [48]. In addition, functional assessments of those multiple HSCs suggested SATB1 expression was important to sustain both self-renewal and multipotency, and that SATB1 expression levels fluctuated in HSCs for the long-term adult hematopoiesis. Thus, SATB1 may be involved in the fundamental mechanisms regulating the epigenetic status of HSCs. Since bivalent histone modifications on stem cell-related and lineage-related genes are involved in the maintenance of attributes associated with stemness [52,53], it would be intriguing to resolve the elaborate network created by epigenetic regulators in HSCs.

**Figure 2:**
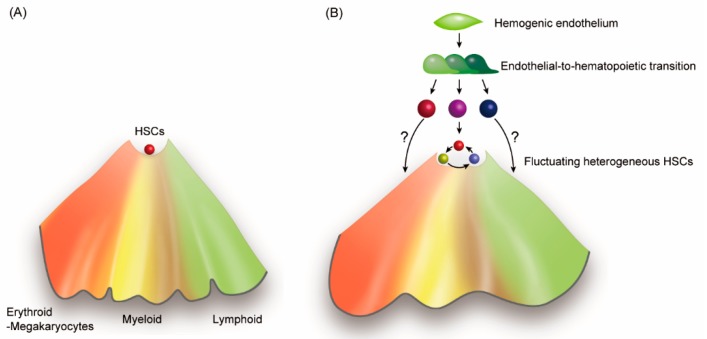
Epigenetic modifications underlie HSC heterogeneity. (**A**) Traditional Waddington’s epigenetic landscape shows the fate decision of HSCs. (**B**) By adding autonomous and/or heteronomous fluctuation of HSCs, the HSC compartment represents diverse types of cells of which lineage tendencies are epigenetically biased. In addition, the figure also includes the notion of lineage-restriction of HSCs during the ontogeny. A recent report indicated that immediate precursors to hematopoietic cells already have their hematopoietic lineage restrictions prior to complete downregulation of the endothelial signature [56].

Waddington’s model clearly described the mechanism by which cell differentiation is affected by the epigenetic landscape (Figure 2A) [54]. However, the mechanism of HSCs to alternate between self-renewal and differentiation at the top of the epigenetic valley, remains elusive. Most of HSCs should be located outside the valley bottom and actively involved in blood cell production, even under homeostatic conditions. In addition, the ontogeny of hematopoietic cells during embryonic and fetal stages consists of multiple pathways that emerge from hemogenic endothelial cells in diverse organs [2,55,56]. Since fetal hematopoiesis produces blood cells prior to the emergence of HSCs in accordance with the developmental requirement, some long-lived lineage-restricted progenitors may contribute to the diversity of the HSC compartment. By exploring a new concept of diversity and fluctuation in HSCs, we may be able to describe the updated model of HSCs self-renewal and differentiation (Figure 2B).

### 2.6. Diversity of the “HSC Niche”

It is important that we understand the environmental regulation of HSC self renewal and differentiation. This knowledge will ultimately be critical for expansion and manipulation of HSCs ex vivo. The bone marrow environment which maintains HSCs and determines their lineage fate involves extracellular matrix (ECM) proteins, secretary factors such as cytokines and chemokines, non-hematopoietic and hematopoietic cellular components, and HSCs themselves.

An early study reported fibronectin, an extracellular matrix (ECM) protein in bone marrow, which supports the proliferation of HSCs in both self-renewing and myeloid-differentiating conditions [57]. Substantial development in multicolor 3-dimensional imaging has provided precise maps of fibronectin distribution throughout the bone marrow [58]. Among major ECM proteins, fibronectin structures, in particular, include a vast network of fibers along which CXCL12⁺ stromal cells and c-Kit⁺ cells extend their cellular processes. The importance of fibronectin as a HSC maintenance factor has also been demonstrated in a recent study which succeeded in long-term ex vivo expansion of mouse HSCs in a chemically defined culture [59]. In this achievement, thrombopoietin, a cytokine capable of expanding HSCs as well as megakaryocyte progenitors and platelet production [60,61,62,63], also played a critical role in combination with fibronectin and c-Kit ligand.

The environment supporting HSCs has been called the “HSC niche”, in which the theory seems to originate from a preconceived idea that HSCs exist in limited coverts in the bone marrow. The concept may have constrained some research in this field to identify specific key cells for HSC maintenance because HSCs themselves are essentially heterogeneous and versatile. Indeed, osteoblasts, endothelial cells, mesenchymal stromal cells, nerve cells, and megakaryocytes have been shown to support the integrity of HSCs. The heterogeneity of the “HSC niche” was comprehensively summarized and discussed in previous review papers [64,65,66]. Given that HSCs change their phenotypes and intracellular metabolism in response to stress or aging, it stands to reason that there are various cell types in bone marrow that influence HSC attributes. Furthermore, extramedullary organs may have their own “HSC niche” to fulfill local hematopoietic demands.

### 2.7. Hierarchy and Holacracy

**Figure 3:**
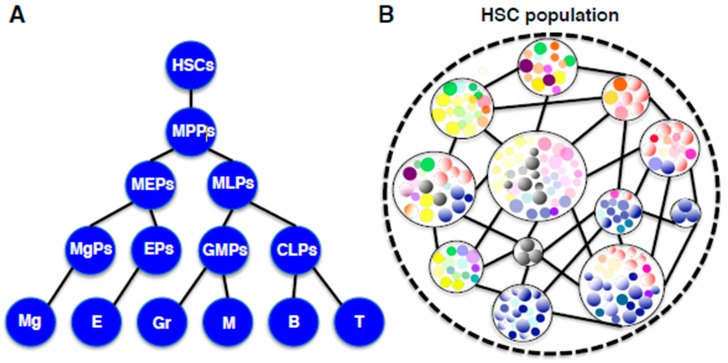
(**A**) A traditional hierarchical diagram of hematopoiesis. In this model, HSCs with self-renewal and differentiation capabilities generate lineage-restricted progenitors in a step-wise manner. Abbreviations: HSCs, hematopoietic stem cells; MPPs, multipotent progenitors; MEPs, megakaryocyte-erythroid progenitors; MLPs, myeloid-lymphoid progenitors, MgPs, megakaryocyte progenitors; EPs, erythroid progenitors; GMPs, granulocyte-macrophage progenitors; CLPs, common lymphoid progenitors; Meg, megakaryocytes; E, erythrocytes; Gr, granulocytes; M, macrophages; B, B lymphocytes; T, T lymphocytes. (**B**) A new unit-networking diagram of HSCs based on recent clonal studies. In this model, the HSC population is comprised of individual HSC-units in which one HSC clone and its derivative self-renewing progenitors represent a spectrum. Every HSC-unit exists in the network consisting of hematopoietic cells, environmental cells, and interactions among them.

Our understanding of hematopoiesis traditionally represents the hierarchical tree diagram, in which HSCs differentiate to lineage-committed precursor cells in a stepwise manner through multipotent, oligopotent and bipotent stages (Figure 3A). Numerous studies have been conducted on this deep-seated notion and have identified the most immature HSCs as well as intermediate progenitors with surface markers [34,35,36,37,38,39,40]. Nevertheless, our hematopoietic system is considered to have evolved during the long process of mammalian evolution to the ideal, which cannot be confined to such a rigid hierarchical organization. As introduced above, lineage fate-committed progenitors with self-renewing activity, comprise the major portion of the HSC population, which have challenged the hierarchy concept of the hematopoietic system [32,33]. In addition, some unilineage-committed progenitors directly derive from multipotent HSCs by bypassing oligopotent stages, suggesting that HSCs are involved in blood cell production in a flexible and dynamic manner [30,67]. Accumulating these observations now reveals the real picture of HSCs in the hematopoietic system. Every HSC is not dormant at the top of the hierarchy, but is fluctuating in both autonomous and heteronomous manners to fulfill an individual function. This system view resembles “holacracy”, a new notion that relates to “sociocracy” in the social science field, and has been developed in organizational control systems [68]. The idea of “holacracy” originated from a “holachy”, which means a “whole”, composed of holons that are self-reliant units, which at the same time play roles as a part of the “whole”. According to this notion, the HSC population can be described as a dynamic ensemble of diverse units which are composed of individual HSCs and their derivatives (Figure 3B). In this case, the “HSC niche” cannot be attributed to some specific milieu but can be viewed as an integral network in which HSCs themselves, as well as surrounding environmental components, are systematically involved.

## 3. Conclusions

In summary, recent findings of active tissue-resident HSCs in the lung and small intestine have challenged the conventional definition of HSCs as being quiescent in the bone marrow. In addition, biophysical computing simulations and comprehensive clonal analysis on HSC behavior have presented their autonomous and/or heteronomous fluctuation, which may underlie the heterogeneity of HSCs and form the foundation of dynamic equilibrium of hematopoiesis. Lineage-restricted progenitors can directly differentiate from HSCs by bypassing hierarchical stages, and some of the lineage-restricted HSCs may arise in early embryos. These findings have challenged the traditional concept of the hematopoietic system as portrayed in a hierarchical tree structure. To characterize the individual “HSC niche” in a network encompassing the hematopoietic system, further experiments quantifying the significance of each cell-cell interaction are required. I believe, however, that it is now time we share an updated theoretical paradigm of HSC biology and hematopoietic organization.
